# Follicular DNA Damage and Pesticide Exposure Among Latinx Children in Rural and Urban Communities

**DOI:** 10.1007/s12403-023-00609-1

**Published:** 2023-11-20

**Authors:** Cassandra Lepetit, Mohamed Gaber, Ke Zhou, Haiying Chen, Julia Holmes, Phillip Summers, Kim A. Anderson, Richard P. Scott, Carey N. Pope, Kirstin Hester, Paul J. Laurienti, Sara A. Quandt, Thomas A. Arcury, Pierre-Alexandre Vidi

**Affiliations:** 1 Laboratoire InGenO, Institut de Cancérologie de l’Ouest, 49055 Angers, France; 2 Department of Cancer Biology, Wake Forest University School of Medicine, Winston-Salem, NC 27157 USA; 3 Sciences Humaines et Sociales, Institut de Cancérologie de l’Ouest, 44805 Saint Herblain, France; 4 Department of Biostatistics and Data Science, Wake Forest University School of Medicine, Winston-Salem, NC 27157 USA; 5 Department of Radiology, Wake Forest University School of Medicine, Winston-Salem, NC 27157 USA; 6 Department of Environmental and Molecular Toxicology, Oregon State University, Corvallis, OR 97331 USA; 7 Department of Physiological Sciences, Oklahoma State University, Stillwater, OK 74078 USA; 8 Department of Epidemiology and Prevention, Wake Forest University School of Medicine, Winston-Salem, NC 27157 USA; 9 Department of Family and Community Medicine, Wake Forest University School of Medicine, Winston-Salem, NC 27157 USA

**Keywords:** Personalized sampling, Pesticides, Farmworkers, Community-based participatory research, Genome instability, Health equity

## Abstract

The intersectional risks of children in United States immigrant communities include environmental exposures. Pesticide exposures and their biological outcomes are not well characterized in this population group. We assessed pesticide exposure and related these exposures to DNA double-strand breaks (DSBs) in Latinx children from rural, farmworker families (FW; *N* = 30) and from urban, non-farmworker families (NFW; *N* = 15) living in North Carolina. DSBs were quantified in hair follicular cells by immunostaining of 53BP1, and exposure to 72 pesticides and pesticide degradation products were determined using silicone wristbands. Cholinesterase activity was measured in blood samples. DSB frequencies were higher in FW compared to NFW children. Seasonal effects were detected in the FW group, with highest DNA damage levels in April–June and lowest levels in October–November. Acetylcholinesterase depression had the same seasonality and correlated with follicular DNA damage. Organophosphate pesticides were more frequently detected in FW than in NFW children. Participants with organophosphate detections had increased follicular DNA damage compared to participants without organophosphate detection. Follicular DNA damage did not correlate with organochlorine or pyrethroid detections and was not associated with the total number of pesticides detected in the wristbands. These results point to rural disparities in pesticide exposures and their outcomes in children from vulnerable immigrant communities. They suggest that among the different classes of pesticides, organophosphates have the strongest genotoxic effects. Assessing pesticide exposures and their consequences at the individual level is key to environmental surveillance programs. To this end, the minimally invasive combined approach used here is particularly well suited for children.

**Supplementary Information:**

The online version contains supplementary material available at 10.1007/s12403-023-00609-1.

## Introduction

Children in agricultural immigrant communities in the United States (US) are at risk for repeated exposures to environmental pollutants. These include pesticides used in fields close to their homes and carried indoors on clothing, and residential pesticides used in housing (Quandt et al. 2004, 2020; Arcury et al. 2007; Tamaro et al. 2018; Hernandez et al. 2019). Children living in urban communities are also exposed to a large number of pesticides, reflecting the ubiquitous presence of currently used and persistent legacy pesticides (e.g., Iglesias-Gonzalez et al. 2020, 2022; Arcury et al. 2021). The emerging picture is that, despite general trends related to location and parental activities, individual exposomes of children are extensive and unique.

Pesticide exposure in children is of particular concern. Their high surface area to volume ratio and immature metabolism lead to higher pesticide doses than in adults (Neri et al. 2006). Pesticides have been shown to alter neurocognitive development in children (Bouchard et al. 2011; Engel et al. 2011, 2016; Rauh et al. 2011, 2012; Bahrami et al. 2022; Dobbins et al. 2022). Epidemiological studies focused on children have also identified associations between chronic exposure to pesticides and pediatric cancers (brain tumors and leukemia) (Ma et al. 2002; Van Maele-Fabry et al. 2013; Cha et al. 2014; Chen et al. 2015; Chevrier and Beranger 2018). Environmental exposures during developmental windows of susceptibility such as early adolescence may also affect health outcomes later in life. Yet, the modes of pesticide toxicities at low, chronic doses are poorly understood (Grandjean and Landrigan 2006). Establishing causality between pesticide exposures and long-term health outcomes, such as cancers and neurological disorders, is challenging due to the long latencies of these diseases. In addition, complex mixtures and doses of pesticides are difficult to reproduce with cell or animal models. Functional biomarkers in exposed subjects provide rapid approaches to predict the impact of environmental exposures on health. Among these markers, DNA damage is a pathological characteristic of cancers and other chronic diseases (Fishel et al. 2007; Kulkarni and Wilson 2008; Ciccia and Elledge 2010; Shiwaku and Okazawa 2015).

Previous studies have assessed DNA damage as a function of pesticide exposures in children and generally found positive correlations. A frequent limitation of these studies is that a single pesticide, or a class of pesticides was monitored and correlated to DNA damage levels. These include organochlorine pesticides (Anguiano-Vega et al. 2020), dichloro-diphenyl-trichloroethane (DDT) and its metabolites (Perez-Maldonado et al. 2006), organophosphate pesticides (How et al. 2014; Sutris et al. 2016), and atrazine (Ruiz-Guzman et al. 2017). The detection of a particular type of pesticide is likely to be associated with exposures to other pesticides that may cause or modulate the measured genotoxic outcome, making it difficult to establish causal relationships.

Noninvasive biosampling is important for studies with children. We developed a sensitive and minimally invasive assay to quantify DNA double-strand breaks (DSBs) in plucked hair follicles (Vidi et al. 2017). We focus on DSBs because these lesions are highly deleterious. When incorrectly repaired, DSBs can cause major genomic rearrangements or mutations that contribute to tumorigenesis (Ciccia and Elledge 2010). Hair follicles contain epithelial cells, which is relevant since most solid tumors are of epithelial origin. Hair follicles also contain stem cells (Gho et al. 2004; Cotsarelis 2006) that give rise to proliferating cells in anaphase hairs (Camidge et al. 2005). Dysfunctional stem cells have been proposed to be at the origin of multiple malignancies. Proliferation occurs in epithelial tissues to replace damaged cells and in response to physiological needs (e.g., the expansion of the mammary gland for lactation). DNA replication in proliferating cells can lead to the conversion of unrepaired single-stranded breaks and oxidative DNA damage into DSBs. From this perspective, hair follicles are interesting compared to other common biosampling sources, such as blood and exfoliated buccal cells, which have few proliferating cells. The hair papilla, at the tip of the follicle, is irrigated by blood capillaries. Hence, follicular cells sense systemic chemicals, as evidenced by hair loss following chemotherapy. Hair follicular cells have actually been used to measure DNA damage induction by anticancer drugs (Fong et al. 2009; Redon et al. 2010) and to monitor DNA breaks induced by ionizing radiation (Tepe Cam and Seyhan 2013; Kudlova et al. 2021). Paired with pesticide biomonitoring, quantification of DSBs in hair follicles may be used to assess genotoxic effects of pesticide exposures in children (Vidi et al. 2017). Here we report the implementation of this concept with Latinx children from the PACE5 (Preventing Agricultural Chemical Exposure 5) cohort.

## Materials and Methods

### Participant Recruitment

All participants were enrolled in the PACE5 study, a community-based participatory research project conducted in partnership between the North Carolina Farmworkers Project (Benson, NC; https://ncfwp.org/) and Wake Forest University School of Medicine (WFUSM). PACE5 is a longitudinal study on the health and cognitive effects of pesticide exposure for Latinx children in rural farmworker (FW) families and in urban non-farmworker (NFW) families. Study protocols and procedures were approved by the WFUSM Institutional Review Board. Written parental permission and child assent were obtained for each participant. Inclusion criteria and participant recruitment are described in Arcury et al. (2021). Exclusion criteria for participation in the PACE5 study included (1) having life-threatening illness such as cancer, (2) prior history of neurological conditions, and (3) physical condition or development disorder preventing the participant from completing neurobehavioral tests or MRIs used in the larger main study. The FW participants were from counties surrounding the town of Benson (NC). The NFW participants were from Winston-Salem (NC). This ancillary study was proposed to 83 PACE5 participants. Participants were seen at the last visit of PACE5 for this ancillary study on follicular DNA damage, between April and November 2021. A $20 incentive was provided for participation to the hair follicle study. Characteristics of the participants are described in the Results section.

### Collection of Hair Follicles

Samples of 5–10 hairs were plucked from the scalp of the participants using flat tweezers. Long hairs were cut to keep only 1–2 cm of shaft from the follicle. Hair follicles were fixed immediately after collection with 4% formaldehyde (Sigma) for 20 min, then kept in phosphate buffer saline (PBS) supplemented with 50 mM glycine and 0.02% sodium azide at 4 °C until analysis. We validated sample storage up to 4 months using a set of hair follicles from a healthy donor (Suppl. Fig. S1).

### Immunostaining and Imaging of Hair Follicles

This procedure was performed as described previously (Vidi et al. 2017) with minor modifications. Briefly, the hairs (fixed immediately after collection) were inspected for presence of the follicle and transferred in a 12-well tissue culture plate containing PBS. The follicles were permeabilized with 0.5% Triton X-100, then washed with PBS. 10% goat serum in immunofluorescence buffer (IF; 130 mM NaCl, 13.2 mM Na_2_HPO_4_, 3.5 mM NaH_2_PO_4_, 0.1% bovine serum albumin, 0.05% NaN_3_, 0.2% Triton X-100, and 0.05% Tween 20) was used for blocking and for dilution of antibodies. Incubations with primary antibodies (overnight at 4 °C) and secondary antibodies (1 h at ambient temperature) were followed by washes in IF, and staining with 4′,6-Diamidino-2-phenylindole dihydrochloride (DAPI) and with fluorescently labeled (AlexaFluor-488) phalloidin. Stained follicles were mounted with coverslips using ProLong Gold Antifade (Invitrogen). Antibodies were against 53BP1 (Abcam, cat# Ab36823, 5 µg/ml), MDC1 (Abcam, cat# Ab50003, 2.3 µg/ml), and γH2AX (Millipore, clone JBW301, 2 µg/ml). Secondary antibodies conjugated with Alexa Fluor dyes AF568 or AF647 (ThermoFisher) were used at 1:500 dilutions. Optical sections of the follicles were taken with a Zeiss LSM880 confocal microscope, using a 40 × water immersion objective (NA = 1.1). Similar regions and tissue depths were imaged for each participant.

### Measure of DNA Double-Strand Breaks in Follicular Cells

Foci of 53BP1 in nuclei of follicular cells were quantified by visual scoring by an investigator blind to the experimental groups and pesticide exposure data. 53BP1 is a DNA double-strand break repair factor that forms distinct microscopic foci at DSBs (Schultz et al. 2000; Bekker-Jensen et al. 2005). We showed previously that the number of 53BP1 foci increases in cells from irradiated hair follicles, and that this increase is radiation dose-dependent (Vidi et al. 2017). 53BP1 foci generally overlapped with foci formed by MDC1 (Mediator of DNA damage checkpoint protein 1), another DSB repair factor (Suppl. Fig. S2A). Concordance of 53BP1 foci enumeration between independent scorers was high (Suppl. Fig. S2B), which validated the quantification of this marker. We obtained follicular DNA damage data for 45 participants (30 FW and 15 NFW). The absence of cellularized follicles was the principal reason for missing DNA damage data for some of the participants, as some hair shafts broke off when pulling the hairs. Between one and seven hairs were analyzed for each participant (median = 3). Values were summarized by averaging the numbers of 53BP1 foci per nucleus, combining each cell in each hair of a participant. The number of nuclei assessed ranged from 40 to 585 (median = 184).

### Deployment of Passive Pesticide Samplers

Silicon wristbands (24hourwristbands) were used to monitor pesticide exposure, as reported previously (Anderson et al. 2014; Donald et al. 2016; Arcury et al. 2021). Briefly, the wristbands were rinsed in deionized water before their deployment to remove particulates (Anderson et al. 2017). The wristbands were then conditioned for three hours in a vacuum oven (300 °C, 0.12 Torr) with periodic nitrogen sweeps, and packaged individually in Teflon bags. The participants were instructed (in the presence of their parents) to wear the wristband for seven consecutive days. The wristbands were actually worn for 6–17 days (median = 8 and 7 days in the FW and NFW groups, respectively). An empty Teflon bag was given to the parents to store the wristband at the end of the sampling period, until laboratory analyses.

### Laboratory Analyses of the Passive Samplers

The wristbands were first rinsed with Milli-Q water and isopropanol to remove particulate matter. Tetrachloro-meta-xylene, decachlorobiphenyl, and PCB100 were used as extraction surrogates. The wristbands were extracted with ethyl acetate, twice 50 ml which were combined and reduced to 1 ml. When analytical interferences had to be removed, solid-phase extraction was performed with acetonitrile on a C18 silica column (Kile et al. 2016). The solvent was exchanged to isooctane prior to injection. All solvents (from Fisher Scientific) were Optima-grade or equivalent. Analytical grade standards (from Accustandard) had purities of 95% or higher. We used an analytical method previously developed and validated for the analysis of pesticides in wristband samplers (Kile et al. 2016; Vidi et al. 2017). The internal standard (4,4′-dibromooctafluorobipheny) was added, and the extracts were analyzed using an Agilent 6890N gas chromatograph with dual micro-electron detectors. For most analytes, a DB-17MS column was used for identification and quantitation whereas a DB-5MS column was used for confirmation. Pesticides with concentrations above the level of detection (LOD) were considered to be present in the samples.

### Pesticide Exposure Measures from Passive Samplers

Measures of pesticide exposure are the presence/absence of 72 pesticides and pesticide degradation products. These were selected based on previous toxicology and exposure studies (Anderson et al. 2017; Harley et al. 2019). The pesticides and pesticide degradation products are from 15 classes (organochlorine, pyrethroid, organophosphate, phenylpyrazole, neonicatinoid, chloroneb, dicarboximide, pentachloronitrobenzene, thiadiazole, dinitroaniline, aniline, triazine, benzenedicarboxylic acid, oxadiazole, and thiocarbamate) and are listed in Table 2.

### Blood Collection to Assess Cholinesterase Activity

Blood samples were collected from a finger stick into capillary tubes. The participant’s skin was cleaned with an alcohol wipe before collection. The amount collected was 2 to 3 drops of blood, with a drop equivalent to 50 μl. Samples were frozen at – 20 °C.

### Laboratory Analysis of Cholinesterase Activity

Total cholinesterase, acetylcholinesterase and butyrylcholinesterase activities were assayed using a modified version of Johnson and Russell’s radiometric method with differential inhibition conditions (Johnson and Russell 1975), as described (Quandt et al. 2010, 2015). Whole blood was diluted with 50 mM neutral potassium phosphate buffer (1:30) immediately after thawing. All measurements were done in duplicates. Diluted whole blood (20 µl) was added to scintillation vials, with either a specific acetylcholinesterase inhibitor (60 µl of BW284C51; 1.5 μM final concentration; (Mirajkar and Pope 2008)) or with the same volume of buffer. This concentration of BW284C51 completely inhibited acetylcholinesterase activity in our assay conditions. The substrate ([3H]acetylcholine iodide, 20 µl; 1 mM final concentration) was added to each vial. After 10 min incubation at 37 °C, the reactions were stopped by addition of acidic stopping mixture (100 μl; pH 2.5), and 5 ml of organic scintillation cocktail was added. The vials were mixed vigorously and counted the following day to allow equilibration of the aqueous and organic phases. Before analyzing participant samples, total hydrolysis of the substrate (Ymax) was determined from a time course assay using a one phase association model (Graphpad Prism 6). Cholinesterase activity in participant samples was then expressed relative to Ymax. Total cholinesterase activity is defined as the activity in the samples without BW284C51. Butyrylcholinesterase activity is defined as the amount of activity in the presence of BW284C51. Acetylcholinesterase activity is defined as the difference in activity between those two conditions.

### Assessment of Sun Exposure

At the time of hair collection, a questionnaire was administered to the parent/guardian to evaluate UV exposure, a potential confounder for DNA damage in hair follicles. The questionnaire assessed how many times the child played outside in the sun in the last week (none; 1–2 times; 3–4 times; ≥ 5 times) and how often hats were worn for sun protection (all of the time, some of the time, or never).

### Measure of Participant BMI

Participant weight (kg) and height (cm) were measured in duplicates and averaged. Body mass index (BMI) was calculated as weight divided by squared height. WHO BMI tables for children (ages 5–19) were used to assign participants to weight categories. BMI measures were taken at the study baseline visit (between March 2018 and May 2019). For a subset of the participants, longitudinal BMI measurements were available and are displayed in Suppl. Fig. S3A, indicating overall stable trajectories with expected increases with age. The fold change in BMI was not different for children from the FW and NFW groups (Suppl. Fig. S3B).

### In Vitro Measurements of DNA Damage

Non-neoplastic HMT-3522 S1 breast epithelial cells were propagated between passages 54 and 60 in H14 medium, as described (Vidi et al. 2013). The cells were transduced to express a fluorescent DSB sensor, namely mCherry fused to the minimal DSB foci-forming region of 53BP1 (mCh-53BP1_ct_; residues 1220–1711). The cells were treated with chlorpyrifos (CPF) for 24 h. The concentration of CPF (1 µg/ml) was based on previous analyses from the CHAMACOS study, which identified up to 1.7 µg/ml of plasma CPF (Huen et al. 2012). In independent experiments, hair follicles plucked from an adult volunteer were treated with CPF diluted in DMEM medium (0.1 and 1 µg/ml), then immunostained for 53BP1 and H2AX. The mCh-53BP1_ct_ signals and immunostained DSB foci were quantified by visual scoring.

### Other Characteristics

Other participant characteristics considered in the analysis are location group (farmworker, non-farmworker), gender (girl, boy), and age (in years). The season in which the hairs were collected is defined as April-June, July–September, and October–November.

### Statistical Analyses

Statistical analyses were done using Graphpad Prism 9 and Stata. Normality of data distribution was tested with the D’Agostino & Pearson omnibus test. If the data did not pass the normality test (at alpha = 0.05), nonparametric approaches were used. The *t* test (or Mann–Whitney test) was used for comparisons between two conditions. ANOVA and Tukey post hoc test (or Kruskal–Wallis and Dunn’s multiple comparisons test) was used for datasets with more than two levels. All statistical tests were two-sided. A *P* value < 0.05 was considered significant. Unless indicated otherwise in the figure legends, datapoints in graphs represent values for individual study participants and summary values are mean ± SD.

## Results

### Characteristics of the Participants

Eighty-three active PACE5 participants were contacted for this study and 70 (84%) agreed to participate. Acceptance rates were comparable for children from farmworker and from non-farmworker families (82% and 86%, respectively). Follicular DNA damage data was obtained from 45 participants (30 FW; 15 NFW). All data shown in this report concern these 45 participants. Loss of participants was either due to collection of insufficient number of hair follicles with cells at their tips (*N* = 12), or to technical issues with the immunostaining protocol (*N* = 13). All participants with available follicular DNA damage data were between 10 and 12 years old, with average ages of 10.7 and 11.1 in the FW and NFW groups, respectively. The proportion of girls/boys was 40/60% in the FW and 47/53% in the NFW group. The body mass index of the FW and NFW children was similar (18.9 ± 2.9 and 19.7 ± 3.5; *P* = 0.58, unpaired t test). The characteristics of the participants are summarized in Table 1.

**Table 1:** Personal and study characteristics of the participants, by location groups

Participant characteristic*	Farmworker	Non-farmworker	Total
Gender of child			
Girl	12 (40.0)	7 (46.7)	19 (42.2)
Boy	18 (60.0)	8 (53.3)	26 (57.8)
Age (years)	10.7 ± 0.5	11.1 ± 0.5	10.8 ± 0.6
BMI (kg/m^2^)	18.9 ± 2.9	19.7 ± 3.5	19.1 ± 3.1
Collection time			
April–June	12 (26.7)	1 (2.2)	13 (28.9)
July–September	12 (26.7)	13 (28.9)	25 (55.6)
October–November	6 (13.3)	1 (2.2)	7 (15.6)
Hairs analyzed per participant	3.7 ± 1.4	2.7 ± 1.1	2.9 ± 1.4
Days wristbands were worn	8.9 ± 2.2	7.0 ± 0	8.3 ± 2.0

*Participants from the PACE5 study with available follicular DNA damage data. Data are presented as *n* (%) and mean ± SD

### DNA Breaks by Participant Location and Time of Collection

**Fig. 1:**
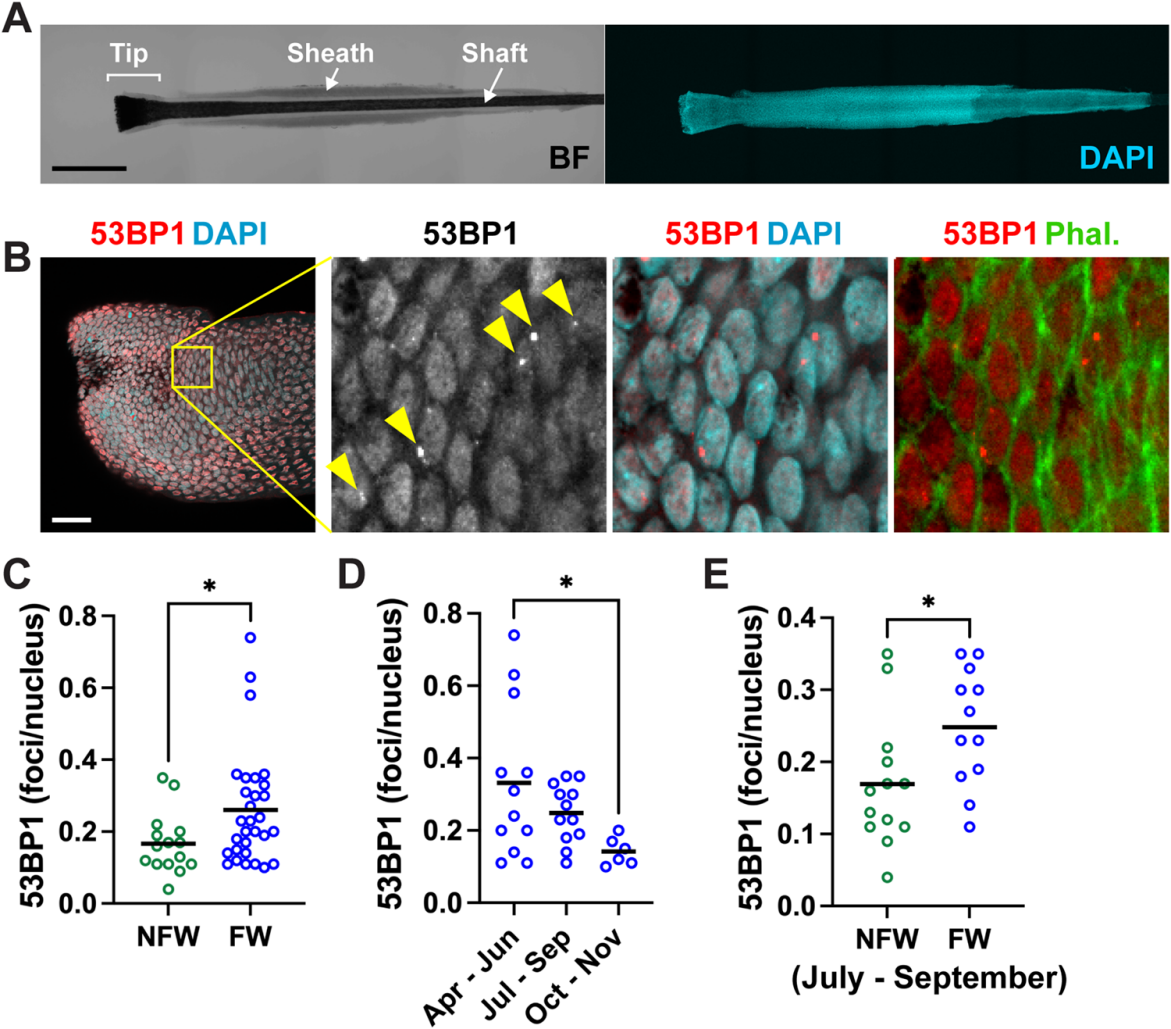
Detection of DNA breaks in hair follicles from Latinx children living in non-farmworker (NFW) and farmworker (FW) families. **A** Low-magnification images illustrating the different parts of a plucked hair follicle (brightfield; BF) and the presence of follicular cells (DAPI). Scale bar, 500 µm. **B** Tip of a hair follicle stained for the DNA double-strand break marker 53BP1. Nuclei were labeled with DAPI, and actin was labeled with phalloidin. Arrowheads indicate DNA damage foci in the enlarged image. Scale bar, 50 µm. **C** Quantification of 53BP1 DSB foci in hair follicles from NFW and FW children. *, *P* = 0.029 (Mann–Whitney). **D** Comparison of DSB levels in FW children at different times of collection. *, *P* = 0.030 (Kruskal–Wallis and Dunn’s multiple comparison test). **E** 53BP1 foci counts in hair follicles collected during the period from July to September 2021. *P* = 0.026 (Mann–Whitney)

53BP1 damage foci were quantified from confocal images taken at the tips of the hair follicles (Fig. 1A–B). FW children had significantly more DSB foci than NFW children (Fig. 1C). Hair collection for the FW children took place between April and November 2021, enabling us to assess seasonal effects. As shown in Fig. 1D, the highest levels of DNA damage were measured in April-June, whereas the lowest levels were measured in October–November. Most (87%) collections from NFW children were done in July and August 2021. We could, therefore, not assess seasonality in the NFW group. We compared DSB levels in the two groups over a similar summertime period (July–September) and again found higher levels of DNA damage in children from farmworker families (Fig. 1E), indicating that the difference between the two groups was not caused by seasonal effects.

**Fig. 2:**
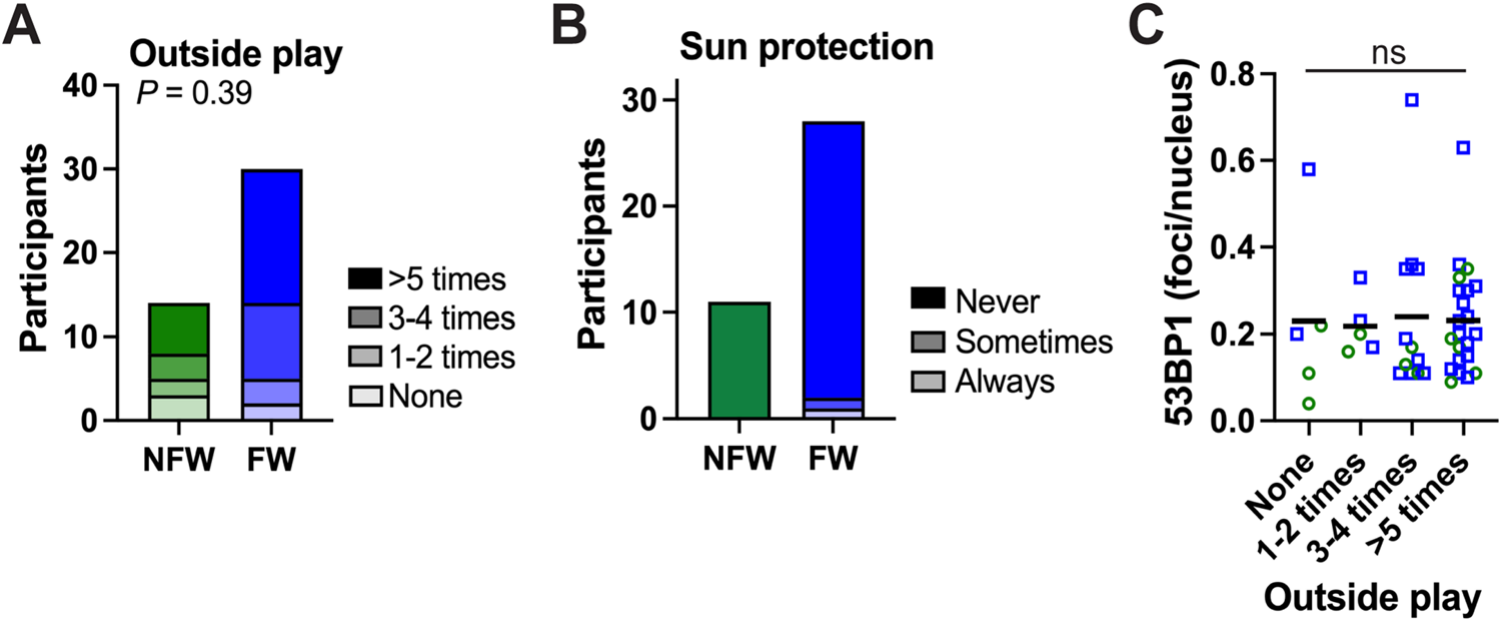
Effect of sun exposure on follicular DNA damage. **A–B** Questionnaire data on how often children from non-farmworker (NFW) and farmworker (FW) families played outside in the sun in the last seven days preceding the hair collection (**A**) and, for those who did play outside, if a hat (sun protection) was worn (**B**). Questionnaire data was missing for one FW participant. Statistical comparison using Fischer exact test. **C** 53BP1 foci counts for the different sun exposure categories. ns, *P* = 0.92 (Kruskal–Wallis). Green circles represent NFW children and blue squares represent FW children

The majority of both FW and NFW children played outside in the sun at least three times during the week preceding hair collection. The FW children played outside more often than the NFW children, but the difference was not significant (Fig. 2A). Except for two participants in the FW group, no one wore a hat for outside play (Fig. 2B). Average follicular DSB counts were not different between sun exposure categories (Fig. 2C). UV damage from the sun is therefore unlikely to have affected DSB levels in hair follicles in this study.

Boys had more DSB foci than girls, but the difference was not significant (0.26 ± 0.17 vs. 0.18 ± 0.09; *P* = 0.34; Mann–Whitney). We also examined whether BMI was associated with DSB levels. Although DNA damage was not statistically different between BMI categories (Suppl. Fig. S3C), DSB levels were higher in obese children compared to the normal weight and overweight groups.

### Pesticide Exposures Detected with Personal Sampling Devices

**Fig. 3:**
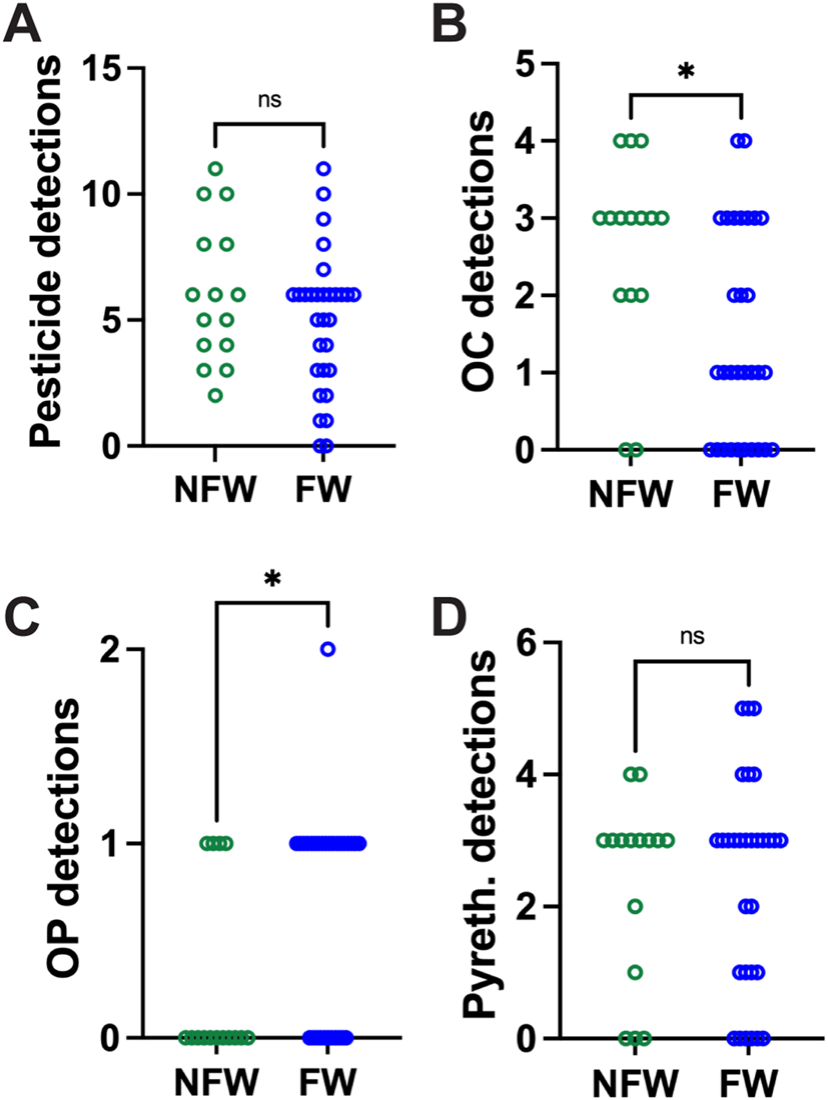
Number of pesticides detected in the wristband samplers worn by children from non-farmworker (NFW) and farmworker (FW) families. The graphs present total detections (**A**), as well as organochlorine (OC) (**B**), organophosphate (OP) (**C**), and pyrethroid (Pyreth.) (**D**) detections. *, *P* < 0.05; ns, not significant (Mann–Whitney)

Pesticides were detected in 43 participants (96%), with 5.3 ± 2.8 (0–11) detections on average. The number of pesticides detected in FW and NFW children was similar (median = 6 in both groups), but the type of exposures was different, with more organochlorine pesticides detections in NFW compared to FW participants, and more organophosphate pesticide detections in the FW than in the NFW group. Differences in exposure to organophosphates between FW and NFW children were stark, with 63% of children in farmworker families exposed to at least one organophosphate pesticide compared to 27% for children in non-farmworker families. Chlorpyrifos was the most frequently detected organophosphate (in 21 participants). Ethion was detected in two non-farmworker participants, whereas dimethoate was detected in one farmworker participant. The number of pyrethroid pesticide detections was similar in both groups. Detections are summarized in Fig. 3 and listed in Table 2.

**Table 2:** Pesticides analyzed and their frequency of detection by location [*n* (%)]

Pesticides and degradation products	Farmworker	Non-farmworker	Total
*Insecticides*			
Organochlorine^a^			
4_4 DDE	1 (3.3)	1 (6.7)	2 (4.4)
Alpha-Chlordane	12 (40.0)	12 (80.0)	24 (53.3)
Gamma-Chlordane	11 (36.7)	12 (80.0)	23 (51.1)
Chlorothalonil	2 (6.7)	1 (6.7)	3 (6.7)
Dieldrin	2 (6.7)	2 (13.3)	4 (8.9)
Heptachlor	0	1 (6.7)	1 (2.2)
Lindane	1 (3.3)	0	1 (2.2)
Mirex	1 (3.3)	0	1 (2.2)
Trans-Nonachlor	10 (33.3)	10 (66.7)	20 (44.4)
Pyrethroid^b^			
Bifenthrin	2 (6.7)	1 (6.7)	3 (6.7)
Cypermethrin	19 (63.3)	9 (60.0)	28 (62.2)
Cis-Permethrin	18 (60.0)	10 (66.7)	28 (62.2)
Trans-Permethrin	18 (60.0)	10 (66.7)	28 (62.2)
Deltamethrin/tralomethrin	1 (3.3)	0	1 (2.2)
Esfenvalerate	5 (16.7)	2 (13.3)	7 (15.6)
L-Cyhalothrin	4 (13.3)	2 (13.3)	6 (13.3)
Organophosphate^c^			
Chlorpyrifos	19 (63.3)	2 (13.3)	21 (46.7)
Dimethoate	1 (3.3)	0	1 (2.2)
Ethion	0	2 (13.3)	2 (4.4)
Phenylpyrazole			
Fipronil	0	1 (6.7)	1 (2.2)
Fipronil-sulfide	5 (16.7)	5 (33.3)	10 (22.2)
Fipronil-sulfone	2 (6.7)	0	2 (4.4)
Neonicotinoid—Acetamiprid	0	0	0
*Fungicides*^*d*^			
Chloroneb	7 (23.3)	4 (26.7)	11 (24.4)
*Herbicides*^*e*^			
Dinitroaniline—Trifuralin	0	2 (13.3)	2 (4.4)
Acetanilide—Metholachlor	0	1 (6.7)	1 (2.2)

^a^Organochlorine insecticides not detected: 4_4 DDD; 4_4 DDT; aldrin; alpha-BHC; beta-BHC; delta-BHC; chlorobenzilate; chloropropylate; endosulfan I; endosulfan II; endosulfan-sulfate; endrin; endrin aldehyde; endrin ketone; heptachlor-epoxide; hexachlorobenzene; isodrin; methoxychlor; o,p’-Dicofol; p,p’-Dicofol; perthane

^b^Pyrethroid insecticide not detected: cyfluthrin

^c^Organophosphate insecticides not detected: chlorpyrifos-methyl; diazinon; ethoprophos; fenitrothion; fonofos; imidan; parathion-ethyl; parathion-methyl; phorate

^d^Fungicides not detected: captafol; captan; etridiazole; iprodione; pentachloronitrobenzene; vincolzolin

^e^Herbicides not detected: alachlor; atrazine; dacthal; diallate I; oxadiazon; pendimethalin; propachlor; propanil; simazine

### Follicular DNA Damage in Relation to Pesticide Exposures

**Fig. 4:**
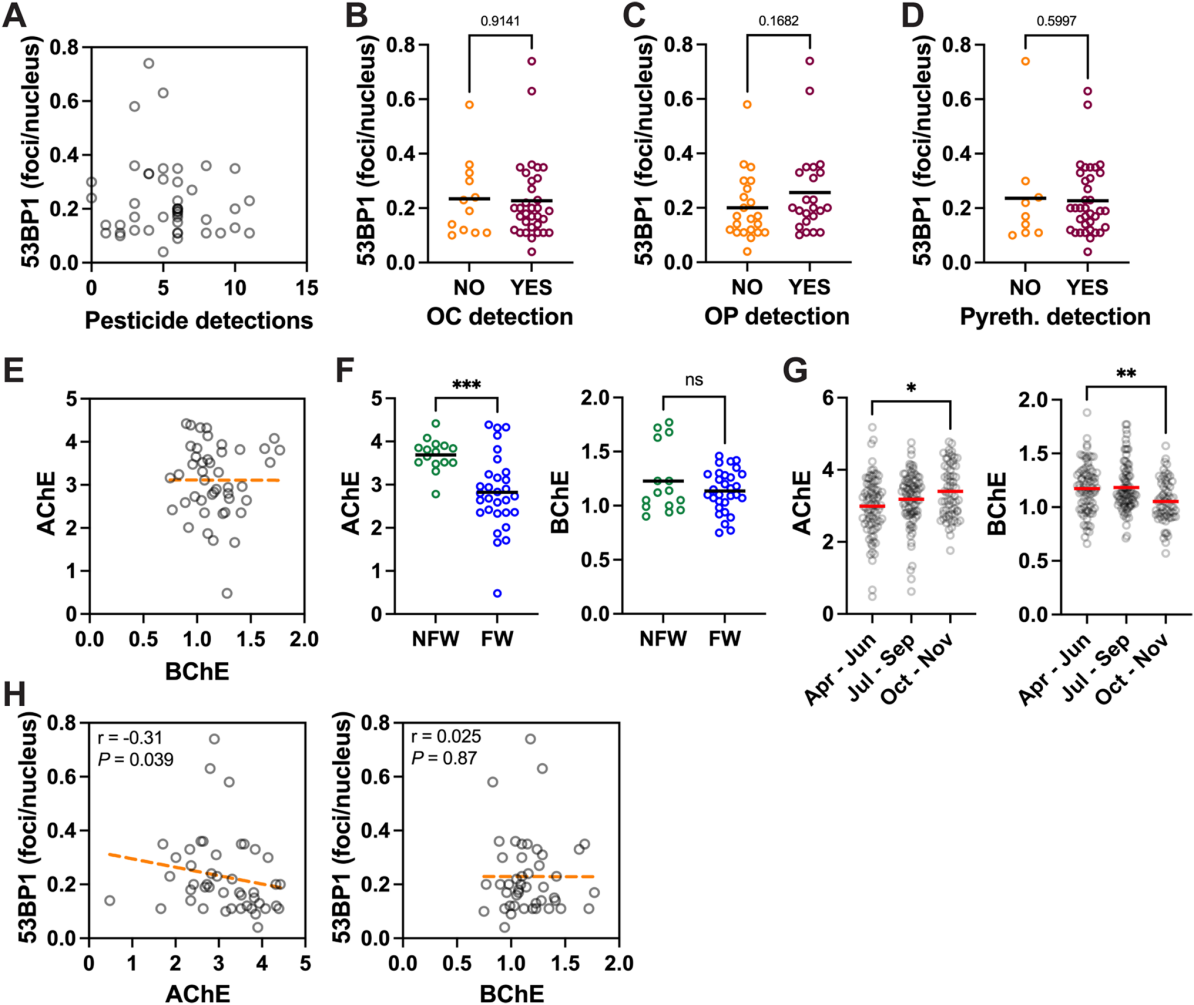
DNA double-strand breaks and exposures to pesticides. **A** 53BP1 foci in hair follicles as a function of the total number of pesticides detected using personal wristband samplers. **B-D** Comparison of 53BP1 foci numbers in participants with or without detection of organochlorine (OC) (**B**), organophosphate (OP) (**C**), and pyrethroid (Pyreth.) (**D**) pesticides. *P* values are indicated (Mann–Whitney). **E** Relationship between acetylcholinesterase (AChE) and butyrylcholinesterase (BChE) activity (µmol/min/ml). **F** AChE and BChE activity by study location, i.e., in children from non-farmworker (NFW) and farmworker (FW) families. ***, *P* = 0.0003; ns, not significant (Mann–Whitney). **G** Seasonality of AChE and BChE activity. Repeated measures (N = 4–9 per participant) are plotted according to the time (month) of measurement. *, *P* = 0.010; **, *P* = 0.0014 (Kruskal–Wallis and Dunn’s multiple comparison test). **H** Association between A/BChE and 53BP1 foci in hair follicles. Spearman’s r coefficients and *P* values are indicated

Overall, there was no correlation between the number of pesticide detections and DSB levels estimated by 53BP1 damage foci counts (Fig. 4A). The number of follicular DSBs were also very similar in participants with and without detection of organochlorine or pyrethroid pesticides. Participants for whom organophosphate pesticides were detected had 30% higher DSB levels compared to those without organophosphate detection (0.26 ± 0.16 vs. 0.20 ± 0.12) (Fig. 4B–D), indicating a possible link between organophosphate pesticides and DNA breaks in follicular cells. To further assess this possible connection, we considered cholinesterase (ChE) depression in blood samples. Acetylcholinesterase (AChE) activity measured in red blood cells and plasma butyrylcholinesterase (BChE) activity did not correlate (Spearman *r* = − 0.10; *P* = 0.51; Fig. 4E). Median AChE activity was 17% lower in children with wristband organophosphate pesticide compared to children without organophosphate pesticide detection, but this difference was not significant (Suppl. Fig. S4). BChE activity was not different in children with vs without organophosphate pesticide detection in their wristbands. AChE activity was significantly lower in FW than in NFW children whereas BChE activity was not different between the two groups (Fig. 4F). Considering seasonality, AChE activity was lowest in April-June, whereas BChE activity was lowest in October–November (Fig. 4G). AChE was significantly anti-correlated with follicular DSB levels (Spearman *r* = − 0.31; *P* = 0.039), whereas BChE and follicular DSB were not associated (Spearman *r* = 0.025; *P* = 0.87) (Fig. 4H). For some participants, there was a time gap between blood and hair collection. Only considering participants with both collection within a month (*N* = 33), the association between AChE and DSB levels was higher than for the PACE5 participants as a whole (Spearman *r* = − 0.39; *P* = 0.023).

### DNA Damage Induction by Chlorpyrifos

**Fig. 5:**
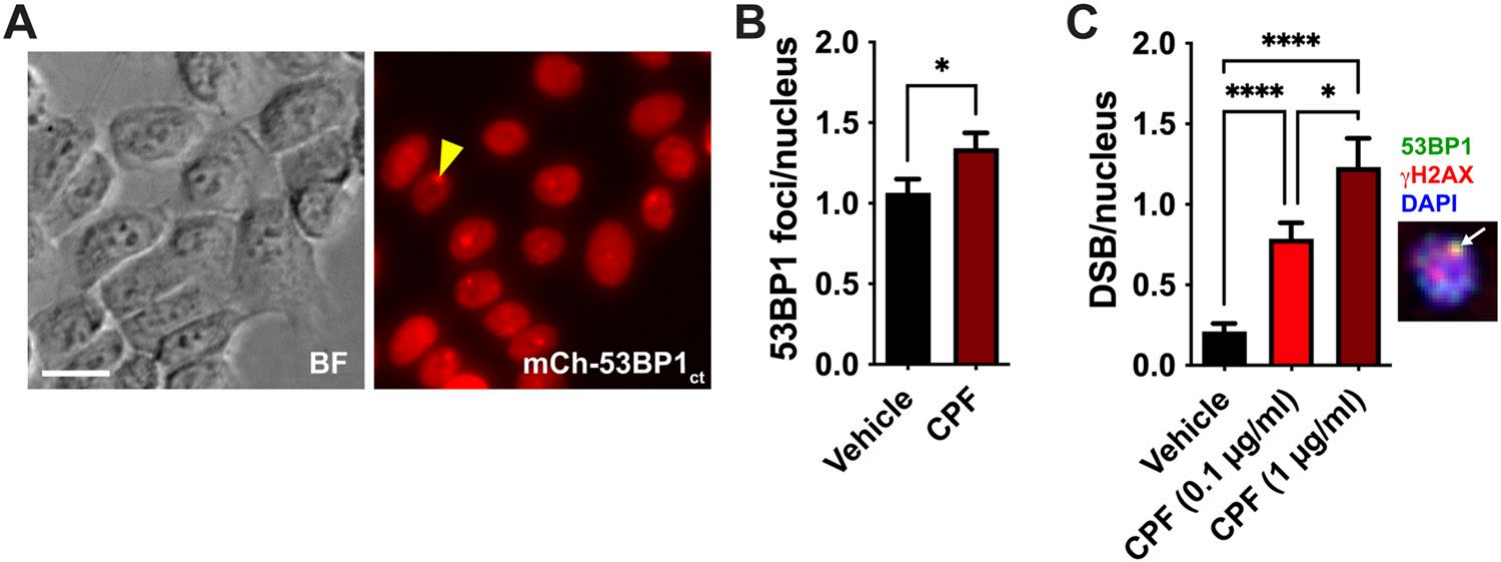
DNA double-strand break induction by chlorpyrifos in epithelial cells. **A** Visualization of DSBs in HMT-3522 S1 breast epithelial cells expressing the minimal focus forming region of 53BP1 fused to the mCherry fluorescent protein (mCh-53BP1_ct_). The arrowhead points to a DSB focus in the fluorescent image. BF, brightfield. Scale bar, 20 µm. **B** Quantification of mCh-53BP1_ct_ in S1 cells treated with vehicle (ethanol) or with chlorpyrifos (CPF, 1 µg/ml) for 24 h. *, *P* = 0.011 (Mann–Whitney). **C** Detection of DSBs in follicular cells after 24 h treatments with vehicle or CPF. DSBs were defined as overlapping 53BP1 and H2AX foci, as illustrated in the inset (arrow). *, *P* < 0.05; ****, *P* < 0.0001 (Kruskal–Wallis and Dunn’s multiple comparison test)

Chlorpyrifos was by far the most frequently detected organophosphate pesticide in our cohort. We therefore tested whether CPF induces DSBs in epithelial cells in vitro. Breast epithelial cells exposed to CPF had significantly more DSB damage foci compared to cells treated with vehicle (Fig. 5A–B). Next, we exposed hair follicles plucked from an adult volunteer to CPF (0.1 and 1 µg/ml) and measured a significant increase in DSB levels for both CPF concentrations compared to vehicle (Fig. 5C).

## Discussion

We implemented a minimally invasive combination approach to quantify DNA damage and pesticide exposures in children. All participants in this community-based participatory research study identified as Latinx, were between 10 and 12 years of age, had comparable (modest) socio-economic status, and lived in North Carolina, either in rural or urban areas. Acceptance to provide hair follicle samples was high (83%); all participants who provided hair follicle samples wore the wristband samplers. Hair collection is a simple procedure that can be done in virtually any setting, including outdoors (Suppl. Fig. S5).

Follicular DNA double-strand breaks were more frequent in children from farmworker families compared to children from non-farmworker families, in agreement with previous studies (Bernardi et al. 2015; Castaneda-Yslas et al. 2016; Kapka-Skrzypczak et al. 2019). Sun exposure is unlikely to have influenced DSB measurements. Outdoor play frequency was not different between FW and NFW children and was not associated with different DNA damage levels. Besides, hair shafts provide a relative UV protection and UV damage causes primarily pyrimidine dimers, not DSBs. The small (non-significant) difference in follicular DSBs between girls and boys may reflect gender-specific differences in genome sensitivities and/or sexual dimorphisms in DNA damage response pathways (Cardano et al. 2022). Gender differences in DNA damage may also reflect the over-representation of boys in the farmworker group, different levels of hair coverage of the scalp (and hence skin protection from pollutants), and differences in behaviors. BMI of FW and NFW children were similar. Obesity therefore cannot explain the rural–urban differences in follicular DNA damage. Yet, our data indicate a possible contribution of obesity to DSB damage in follicular cells, which deserves future attention.

Seasonality of DNA damage levels in children from farmworker families may be due to different pesticide use or different activities (indoor/outdoor play, school/vacation, etc.) at different periods of the year. Our questionnaire on sun exposure was too rudimentary to address the latter possibility. Some organophosphate insecticides, including phorate, acephate, and chlorpyrifos (before its ban in the US in 2021) are used in spring and early summer on immature plants. Paradoxically, pesticide detections (including organophosphates) in wristbands were lower in spring than in fall (Arcury et al. 2023), yet AChE depression was highest in April-June (Fig. 4G). Seasonal effects on AChE activity (as well as the different seasonality of AChE and BChE) corroborate our previous measurements in adult Latinx farmworkers (Quandt et al. 2010, 2015). AChE and BChE activities did not correlate in this study. A previous study by Strelitz et al. (2014) also found no positive correlation between AChE and BChE but rather a weak negative correlation. The absence of positive correlation may (at least partly) be explained by the fact that different organophosphate pesticides have distinct inhibitory potency for AChE and BChE (Cadez et al. 2021) and the persistence of the inhibition by organophosphate pesticides is different for the two enzymes, with shorter inhibition of BChE compared to AChE (Kamel and Hoppin 2004).

The higher organophosphate exposure of FW compared to NFW children reported here based on wristband samplers and cholinesterase depression mirrors the measurements from the entire PACE5 cohort (Arcury et al. 2021), and other environmental and pesticide urinary metabolite measurements from previous studies (Quiros-Alcala et al. 2011; Quandt et al. 2015; Arcury et al. 2016, 2018; Tamaro et al. 2018). Among all the classes of pesticides detected by the wristband samplers, only organophosphates were associated with a modest increase in DNA damage. The association between DNA damage and AChE depression was much stronger, indicating that assessment of internal exposures may better predict biological outcomes than monitoring of external exposures. Wristband sampler measurements correspond to the bioavailable exposure rather than dose, which depends on absorption and metabolism, potentially different between participants.

Although we cannot conclude causality between organophosphate pesticide exposures and follicular DNA damage with our study design, all of our measurements point in this direction and the multiple associations are compelling. Our results are consistent with recent findings of the ORGANIKO study that documented correlations between oxidative DNA damage and chlorpyrifos metabolite detections in the urine of children in Cyprus (Makris et al. 2022). CPF induces reactive oxygen species, oxidative DNA damage and DNA strand breaks (Bagchi et al. 1995; Rahman et al. 2002; Li et al. 2015; Kopjar et al. 2018). It was by far the most frequently detected organophosphate pesticide in our cohort. We measured significantly higher DSB levels in follicular cells exposed in vitro to CPF compared to unexposed cells. In these experiments, CPF doses were more than ten times lower than the highest concentrations detected in plasma from mothers and newborn children from the CHAMACOS cohort (Huen et al. 2012). Mechanistically, oxidative DNA lesions caused by organophosphates may degenerate into DNA double-strand breaks, especially in actively dividing cells, such as those at the tip of hair follicles. This mechanism may, however, not fully explain the connection between organophosphate pesticides and DNA double-strand breaks since other classes of pesticides (such as organochlorines) also induce reactive oxygen species and single-strand breaks in vitro and in vivo (e.g., Bagchi et al. 1995). DNA adducts and strand cross-links formation from direct DNA interactions of organophosphates and their metabolites, and indirect effects on gene expression which may affect the DNA repair potential may also contribute to DNA double-strand break accumulation upon organophosphate exposure (Prathiksha et al. 2023).

This study had limitations. The number of participants was relatively small, with missing data for some participants. The latter will be remediated in future studies by increasing the number of hairs collected. The timing of the wristband and hair collections could not be matched perfectly for all participants. For some participants (11/45), ≥ 30 days elapsed between the two collections, which may have affected the analyses. We do not exclude the possibility that additional factors such as personal care and cosmetic products may influence DSB levels in hair follicles. We are also aware that the detected levels of DSBs depend on both the rates of DNA damage and repair. Inter-individual variability in DNA repair capacity likely contributed to the differences in 53BP1 foci densities between participants. Finally, not all organophosphate pesticides were measured in the wristbands and cholinesterase depletion cannot be attributed to a specific chemical compound, which may in part explain the lack of significant association between the two measures.

In conclusion, our study points to rural disparities in pesticide exposures and follicular DNA damage in Latinx children. Future studies combining monitoring of pesticide exposures and (follicular) DNA damage are needed to (1) assess the contribution of individual pesticides (or pesticide mixtures) to genotoxicity, (2) determine if the findings reported here apply to other child populations, and (3) evaluate the impact of socio-economic status on pesticide exposures and their DNA damaging effects.

### Supplementary Information

Below is the link to the electronic supplementary material.

Supplementary file1 (PDF 5626 KB)

## Data Availability

The data generated during this study are included in this article. Imaging datasets from this study are available from the corresponding author on reasonable request.
