# Supplementary material for: Follicular DNA Damage and Pesticide Exposure Among Latinx Children in Rural and Urban Communities
Source: Expo Health. 2023 Nov 20;16(4):1039–52. doi: 10.1007/s12403-023-00609-1 (PMC11362388; doi:10.1007/s12403-023-00609-1)
Supplement: Supplementary file 1 — Supplementary file1 (PDF 5626 KB) [file 12403_2023_609_MOESM1_ESM.pdf]

SUPPLEMENTARY MATERIALS

Follicular DNA damage and pesticide exposure among Latinx children in rural and urban communities

Cassandra Lepetit *et al*.

Supplementary figures S1 – S5

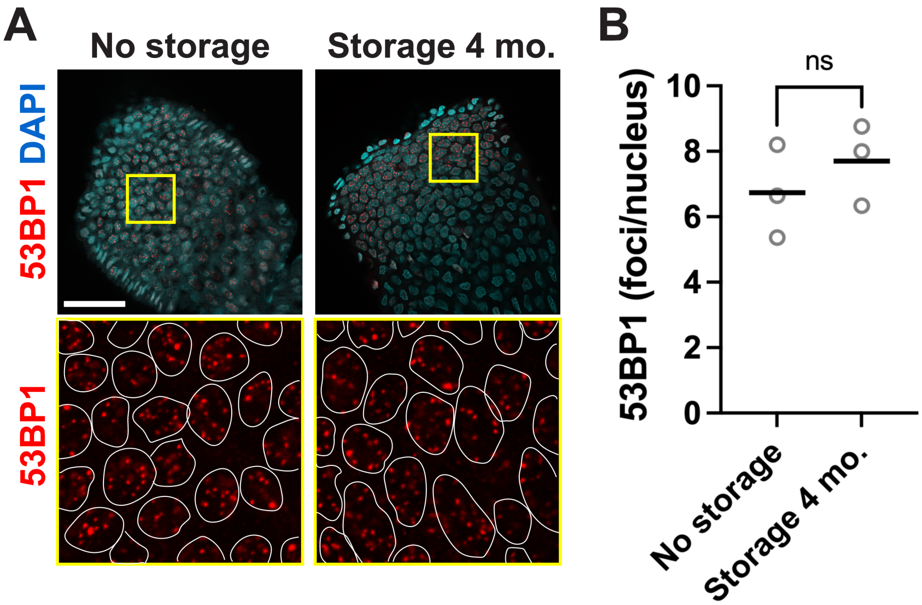

Figure S1. Storage of plucked hair follicles. Hair follicles collected from an adult volunteer were irradiated (3 Gy) with a ^{137}Cs irradiator to induce DSBs, left to recover for 1h at 37°C in DMEM medium, fixed, and stored at 4°C. **A** Comparison of 53BP1 immunostaining, either directly after collection (no storage) or 4 months after collection. Nuclei were stained with DAPI. Enlarged areas with 53BP1 signals are shown at the bottom, with contours of nuclei (white lines). Scale bar, 50 µm. **B** Quantification of 53BP1 damage foci. Symbols are averaged values for individual hairs. ns, not significant (t-test).

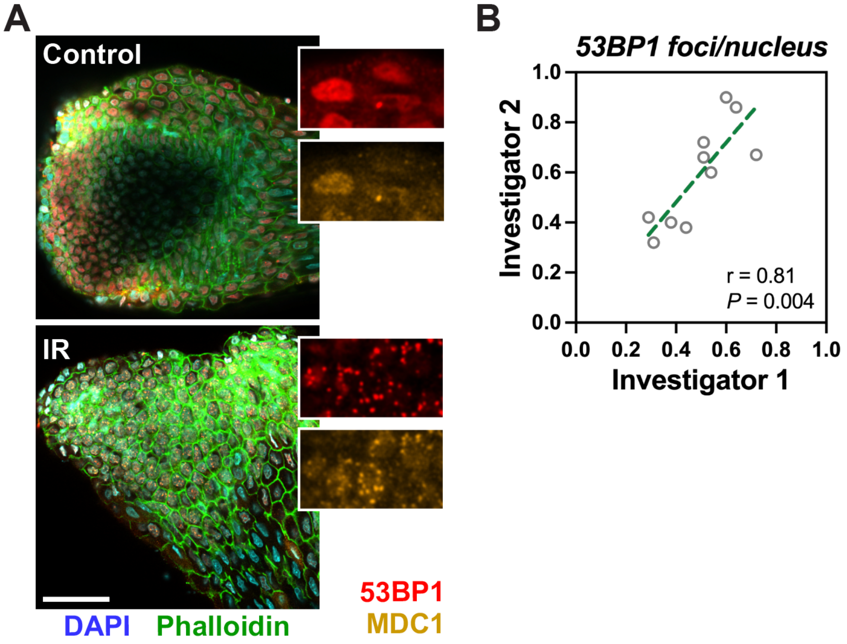

Figure S2. Validation of DSB detection in plucked hair follicles. **A** Dual immunostaining for 53BP1 and MDC1 in untreated (control) or irradiated hairs. Phalloidin staining was used to help delineate the cells. Scale bar, 50 µm. **B** Concordance between independent scorers in the quantification of 53BP1 foci in subset of the participant's hairs. r, Pearson's correlation coefficient.

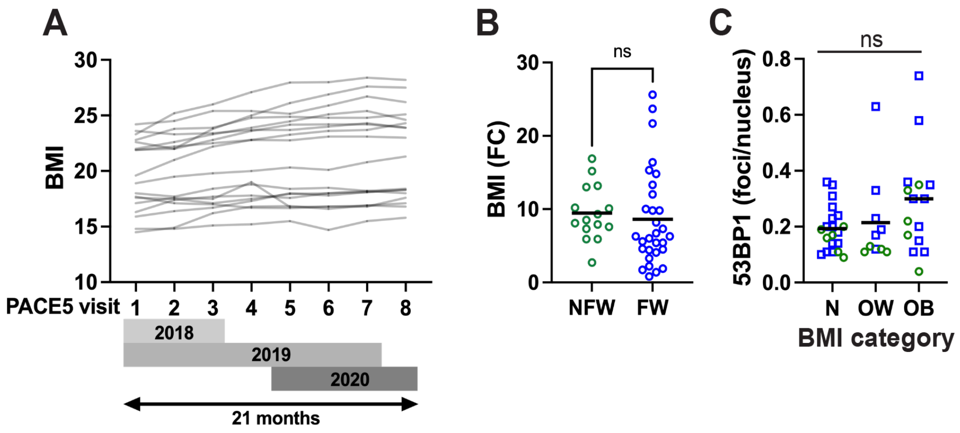

Figure S3. Effect of BMI on follicular DNA damage. **A** BMI trajectories for the first eight quarterly PACE5 visits (2018 - 2020), available for a subset of participants. Hair follicle collection was performed at the last PACE5 visit in 2021. **B** Maximal fold change (FC) in longitudinal BMI measurements for non-farmworker (NFW) and farmworker (FW) participants. **C** 53BP1 foci counts for participants with normal weight (N), overweight (OW), and obesity (OB). Weight categories were assigned based on BMI measured at the first PACE5 visit. ns, $P = 0.23$ (Kruskal-Wallis). Blue squares represent FW children and green circles represent NFW children.

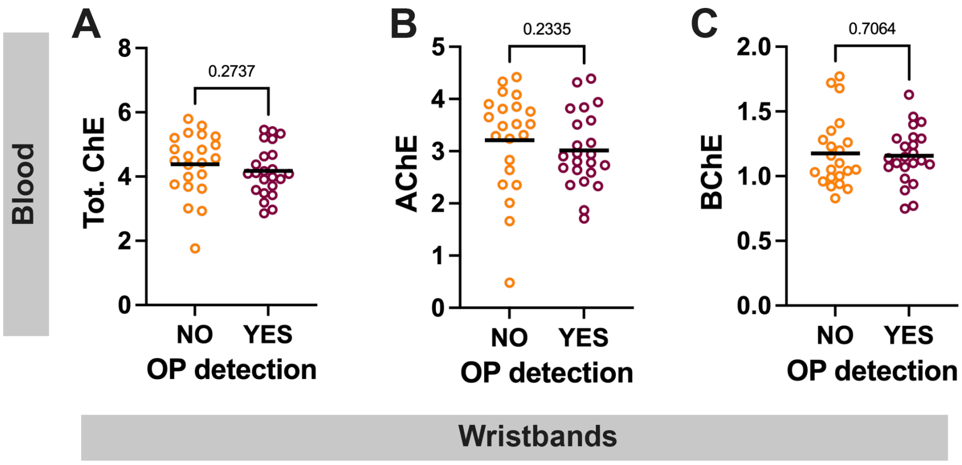

Figure S4. Total cholinesterase (**A**), acetylcholinesterase (**B**), and butyrylcholinesterase (**C**) activities (μmol/min/ml) in participants with or without organophosphate (OP) pesticide detections in their wristband samplers.

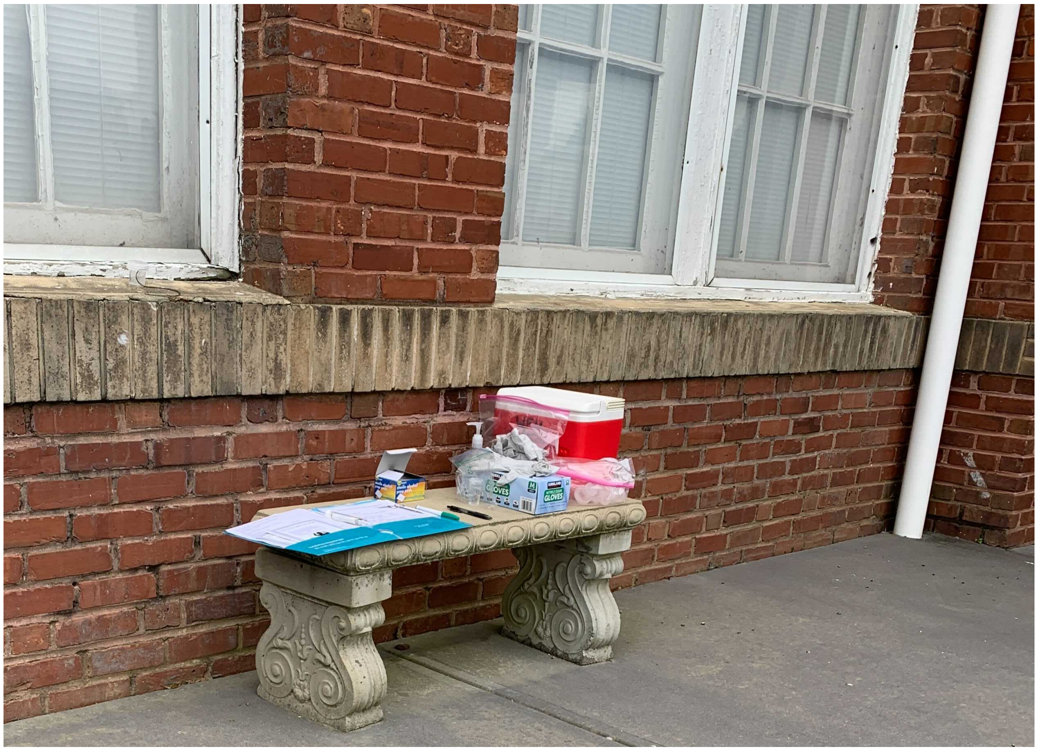

Figure S5. Hair follicle collection site in Benson County, NC. An outdoor location was selected to mitigate COVID-19 infection risk in April 2021.
